# The role of virtual consultations in cancer genetics: challenges and opportunities introduced by the COVID-19 pandemic

**DOI:** 10.1038/s44276-023-00009-7

**Published:** 2023-08-02

**Authors:** Melody G. Redman, Vernie Aguda, Rhys Dore, Jen O. Lim, Beverley Speight, Terri P. McVeigh

**Affiliations:** 1 Yorkshire Regional Genetics Service, Chapel Allerton Hospital, Chapeltown Road, Leeds, LS7 4SA UK; 2 Centre for Medical Education, School of Medicine, Cardiff University, Neuadd Meirionnydd, Cardiff, CF14 4YS UK; 3 Royal London Hospital, Barts Health NHS Trust, Whitechapel Road, London, E1 1BB UK; 4 Department of Pathology, University of Cambridge, Tennis Court Road, Cambridge, CB2 1QP UK; 5 East Anglian Medical Genetics Service, Cambridge Biomedical Campus, Box 134, Level 6, Addenbrooke’s Treatment Centre, Addenbrooke’s Hospital, Cambridge, CB2 0QQ UK; 6 Cancer Genetics Unit, Royal Marsden NHS Foundation Trust, London, SW3 6JJ UK

## Abstract

The COVID-19 pandemic changed the delivery of healthcare within the United Kingdom. A virtual model of care, utilising telephone and video consultations, was rapidly imposed upon cancer genetics teams. This large-scale change in service delivery has led to new opportunities that can be harnessed to improve patient care. There is a clear potential to mitigate geographical barriers, meet increasing patient expectations of implementing virtual consultations, reduce hospital carbon footprints, and decrease hospital costs while increasing efficiency. However, there are also significant challenges introduced by this model of care. Virtual healthcare consultations introduce another new level of digital exclusion for patients and clinicians. There are also potential challenges for maintaining patient confidentiality, and limited utility in circumstances where a physical exam may be warranted. For clinicians, there may be impacts on empathetic responses delivered and challenges in workflow and workload. Virtual consultations are likely to continue being a feature of cancer genetics services. A flexible approach is needed to allow for virtual and traditional models of care to work together and best meet patients’ needs. Cancer genetics services should harness the opportunities provided by virtual processes to improve patient care, whilst collaborating with patient groups and other stakeholders to carefully examine and address the challenges that virtual consultations introduce.

## Background

The COVID-19 pandemic changed the delivery of healthcare as we know it. One mantra became quickly engrained in the public consciousness: ‘Stay at home. Protect the NHS. Save lives’ [1]. The traditional model of care within genetic services involves an in-person appointment with a Clinical Geneticist doctor or Genetic Counsellor (hereafter referred to as “clinicians”). Usually, in-person consultations were arranged for all first appointments and many follow up appointments, with common scenarios in the genetics clinic involving discussions about diagnostic and predictive genetic testing, family history risk, surveillance, risk reduction and reproductive planning. Early in 2020 it became clear to policymakers, National Health Service (NHS) services, and the public that bringing individuals to physical outpatient appointments should only occur when necessary. This led to a rapid re-organisation of how clinical services needed to be delivered.

Cancer services and surgical treatments were significantly impacted by the COVID-19 pandemic [2], and the waiting time for cancer care in the UK was already an area of concern before the COVID-19 pandemic [3]. It was critically important that clinical genetics did not provide an additional delay for these patients. This caused wide scale implementation of virtual consultations accessed from the patient’s personal device at home to be rapidly adopted, in contrast to the slow implementation of digitalisation in other areas, such as electronic health records [4].

Virtual delivery of care was not a new concept to cancer genetics. A pilot study of telephone and video consultations was undertaken by the Cancer Genetics Service for Wales two decades ago, in 2000 [5]. At that time, concerns amongst clinicians largely focussed on challenges in adopting their typical communication style, as non-verbal cues could not be as easily picked up [6]. However, the authors noted potential promise for the virtual model of care as a way to manage increasing service demand, as there were high levels of patient satisfaction [6].

Prior to the COVID-19 pandemic, there had also been growing pressure for policymakers to introduce virtual consultations (VC) in other parts of healthcare, particularly in general practice [7]. The NHS Long Term Plan in January 2019 set out an aim for every patient to be able to access VC for appropriate appointments by 2024 [8]. However, uptake in the UK had been variable [9]. In primary care, it was felt that VC was suitable on occasions when physical examination was not required and when significant medical complexity was not anticipated, such as administrative appointments and reviews of chronic illnesses [7].

A 2012 systematic review explored the role of VC in genetic consultations from 12 studies conducted in the western world [10]. The authors reported generally high levels of patient satisfaction when utilising VC [10]. However, limitations of sample sizes made the generalisability of these findings difficult [10]. There was also a lack of reporting additional outcomes, such as the impact of receiving a diagnosis through VC, that will still need to be explored [10].

In March 2020, the UK entered lockdown due to the COVID-19 pandemic. Pressure quickly mounted to limit non-essential travel, and hospitals sought urgent ways of limiting interaction with large cohorts of patients. NHS England and NHS Improvement offered £20,000 to NHS trusts (NHS organisational units) to enable them to implement VC [11]. By June 2020, around 170 NHS trusts had introduced a VC platform called *Attend Anywhere* [12], which could be accessed on mobile phones, laptops, or other devices [11]. Around 40 trusts introduced alternative VC solutions [11].

Virtual consultations were crucial for enabling provision of cancer genetics services to continue throughout the COVID-19 pandemic. Amidst this context of rapid service change during the COVID-19 pandemic, there is much to reflect upon regarding the opportunities and challenges of virtual consultations in cancer genetics services.

## Opportunities

### Addressing geographical inequalities

One of the notable benefits of the introduction of VC is that it may help alleviate geographical barriers faced by patients accessing cancer genetics services. Due to their specialist nature, these services routinely cover large geographical areas, meaning patients in their catchment area are often many miles from their nearest cancer genetics centre. These centres are also typically situated within metropolitan areas, or close to large academic institutions, placing patients who live in rural areas at a disadvantage for accessing this level of care. Patients may incur significant travel costs when attending in-person appointments, which may be compounded by taking time off work or school to attend appointments during regular work hours.

In 2020, only 3% of UK households did not have internet access [13], whereas 23.9% of households in England do not have a car (this figure becomes 20% if London is excluded) [14]. These crude estimates have many limitations and do not reflect other important factors such as digital literacy or access to public transport. However, they do highlight that generally, households may be more equipped to access virtual consultations, than to travel for a distant in-person appointment.

### Economics and efficiency

One 2016 study compared the time and cost per patient between virtual genetic counselling and traditional in-person genetic counselling for cancer genetics appointments in the Netherlands [15]. They estimated a 7.6% time saving for virtual genetic counselling, resulting in a 10.2% cost saving (€361.22 for in person versus €324.26 for virtual genetic counselling) [15]. This was mainly due to time saved from not having to travel to deliver an in-person clinic. While this may not translate into exact figures for the NHS given differences in service set-up, the reduction in transportation costs and the more efficient employment of clinicians’ time likely mean that there will be cost savings. In addition, virtual interpreting services may save costs and be more readily accessible than in-person ones. With the increasing demand for clinical genetics services and workforce challenges in delivering them [16], efficiencies are important to consider.

The adoption of virtual processes in one area can also result in efficiencies arising in other areas. One example (though this may vary between different services) is that for services relying on paper notes, if a patient does not attend an appointment but a clinician is still based at their desk rather than a distant outpatient clinic, it may be easier to manage other urgent work and responsibilities. In addition, well but self-isolating clinicians can continue to deliver appointments from home using virtual tools [17]. This flexibility may also be helpful for clinicians in other circumstances.

### Meeting patient expectations

As the world around us moves to a more virtual environment and technology becomes further integrated into our daily lives, some patients may expect their healthcare to follow suit. The financial toxicity of a cancer diagnosis and cancer treatment is particularly acute in younger patients of working age, in whom a diagnosis of heritable cancer predisposition is more likely than those diagnosed at older ages, and is exacerbated by loss of hours/days off work [18]. Virtual consultations may enable patients to take less time off work or education, join from the comfort of their own home, or potentially limit the impact on their caring responsibilities. Patients who are currently undergoing cancer treatment may already be attending multiple appointments and may be immunocompromised, so joining an appointment from the safety of their home may be preferable. In addition, waiting in a virtual ‘waiting room’ may afford privacy that a hospital outpatient department does not.

A systematic review in 2021 identified that live synchronous videoconferencing (called ‘telegenetics’ by the authors) had similar psychosocial outcomes, patient satisfaction, and genetic knowledge compared to in-person appointments [19]. They included 13 studies from England, USA, Australia, and the Netherlands [19]. However, the studies were small and subject to at least moderate risk of bias [19].

Before the COVID-19 pandemic, patients referred to a hereditary cancer testing clinic showed low acceptance of virtual appointments with 31% reporting they would accept a virtual pre-test appointment, and 34% reporting acceptance of a virtual results disclosure appointment. After the initial lockdown, these patients were reassessed, and their reported acceptability rates had risen to 92% and 85% respectively [20]. In the 2012 review, it was observed that patients were more pleased with the rapport built compared to the clinical genetics professional [10].

### Carbon footprint

Virtual models of healthcare in other contexts have been shown to reduce healthcare’s carbon footprint, largely from the associated reduction in transport [21]. This fits within the NHS multi-year carbon net zero plan, which includes an aim to deliver care at or close to home [22].

### A family approach

Individuals undergoing genetic testing will often request the presence of their child, parent or sibling at the consultation, for support as well as for their information. Virtual platforms such as *Attend Anywhere* [12] allow the merging of VCs, making it possible to counsel family members together, even if they are in different locations or even different countries. This may not be appropriate in all circumstances and would need to be approached sensitively, whilst upholding all privacy and confidentiality regulations. However, the potential to connect members of a family regardless of physical distance may be advantageous in certain circumstances.

### Challenges

#### Virtual access to genetics services

The 2019 UK Consumer Digital Index found that 22% of the population (11.9 million people) do not have the digital skills needed for everyday life in the UK [23]. This is expected to improve but is still estimated to remain at 8% of the UK population in 2030 [23]. Groups that have been identified as being at a disadvantage include people with a disability, people in lower socioeconomic groups, people in rural areas, and people whose first language is not English [24]. It has already been demonstrated that patients of minority ethnicities are less likely to access genetic testing [25], and one study found that women from a minority ethnic background were the least likely to complete genetic testing during telephone genetic counselling [26]. Digital literacy is not just dependent on access to the internet, but also on skills, confidence and motivation [27]. A study on video and telephone consultations in a primary care setting found that roughly one in four patients reported experiencing technical problems [7].

While virtual care may mitigate geographical barriers some patients face, it is important to collaborate with patients to develop virtual platforms that minimise any digital barriers imposed by this model. Improving the accessibility of cancer genetics services during the ongoing COVID-19 pandemic and beyond may require a mixed approach of virtual and physical appointments.

#### Clinician digital literacy

Similar to patients, clinicians may show a wide range of digital literacy. Some clinicians may feel more comfortable to ‘screen share’ and present drawings and diagrams over VC, which may aid in the explanation of genetic concepts to patients. On the other hand, some clinicians may feel that virtual consultations limit their ability to explain concepts.

In reality, compared to implementation of genomic technology, the NHS has lagged behind in the implementation of digital technology in practice, with a slow transition to electronic notes and often outdated hardware and software and slow internet [28]. Internet connectivity can interrupt the flow of conversation. This may greatly disadvantage patients who rely on sign language, as an unstable video connection makes it very difficult to interpret signs. One 2016 study found that over half of genetic counsellors experienced technical problems during their consultations [15]. It is important to provide the resources and information technology (IT) infrastructure cancer genetics staff need to deliver a virtual service. Data handling procedures will also need to be regularly updated to ensure they comply with all relevant legislation and guidelines.

Some of the operational difficulties around VCs stem from the fact that many commonly used platforms are designed for videoconferencing rather than medical video consultations. Services should ensure they assess the limitations of the chosen platform and consider switching to a more suitable alternative if necessary. Features of platforms designed specifically for VC may include a queue system displaying the patient’s position if there is a wait, allow convenient taking of notes or viewing of test results alongside the video feed, and facilitate three-way communication for another clinician or interpreter [29].

General advice is available for the management of remote consultations [30], but introducing specific advice and training for those undertaking virtual cancer genetics services may be beneficial.

#### Clinical examination

VC limits the ability of the clinician to perform a physical examination. This may sometimes be necessary in cancer genetics consultations when an underlying cancer-predisposition syndrome has relevant physical manifestations. There may be some approaches to address this. For example, head circumference measurement is important in suspected PTEN Hamartoma Tumour Syndrome [31]. Self-measurement of head circumference is generally a reliable technique [32]. However, the presence of other features, such as oral mucosal papillomatosis, may affect eligibility for testing [31]. Some patients may be able to send clinical photographs via email. However, for some referrals, an in-person clinical assessment may be preferable.

Consultations for referrals where a clinical examination is likely to be unnecessary may be more suited to VC. For example, genetic counsellors would not typically be expected to examine a patient for skin features of cancer predisposition syndromes. There is an important role for triage and clinical discretion to identify which consultations may be most appropriate for VCs. This may be difficult to select based on limited information at triage of the initial referral into the genetics service, with potential for syndromic features to be missed. There may also be possibilities to introduce technology for secure integration of photographs submitted by patients via mobile applications, as has been utilised by dermatology services in South East England [33].

#### Clinician experience

While understanding the patient experience is very valuable, it is also important to consider the experience of clinicians delivering the consultations. One small study found a reduction in genetic counsellor satisfaction when using VC over 8 months [15]. However, a study in Australia found that genetics practitioners were satisfied with a VC approach and they felt the advantages outweighed the disadvantages [34]. It is important that the cancer genetics workforce feel valued and satisfied in their work, so this area needs to be monitored.

#### Coercion and confidentiality

When a patient joins a video consultation, or talks over the telephone, the clinician has limited influence over the patient’s surrounding environment. The four walls of a clinic room usually provide confidentiality, safety, and absence of distraction. Sometimes patients who join for a consultation have a flatmate in the room next door, family members in the background, or the distraction of a work environment around them. This may inhibit the patient’s ability to speak freely. Also, it is sometimes unclear if there are influences or pressures from other people to proceed with testing. The patient may also be in an inappropriate environment, such as driving a car. As far as is possible, the clinician needs to remain sensitive to the patient’s surroundings in these situations. Video appointments may provide contextual cues about the patient’s life and home environment [29]. Clinicians should also take care to ensure that information conveyed virtually is not inadvertently shared with others who may physically be in the same room as the clinician or patient.

#### Digital empathy

Bearing difficult news over the telephone or through a digital screen limits the type of empathetic responses that can be provided. However, the effect of this may vary from patient to patient. For example, some patients may prefer to hear that they carry a *BRCA2* pathogenic variant over a VC in the comfort of their own home and potentially surrounded by their support system. However, conveying empathy in virtual consultations has its own challenges and limitations [35]. This may, in turn, influence both patient and provider experience and thus requires further study. Decisions for whether subsequent appointments should be virtual or in-person should be a collaborative process, allowing for both provider discretion and patient choice.

One study found that an in-person cancer genetics appointment versus VC demonstrated no significant difference in the reduction of self-reported anxiety and depression after in-person or video genetic counselling [36], suggesting that virtual platforms may not be inherently introduce barriers to empathy and emotional outcomes.

#### Video versus telephone consultations

Virtual consultations include both video and telephone consultations. Consultations may initially be planned as video, but might become telephone consultations due to technical difficulties. In addition, patients may opt for a telephone appointment above a video appointment, for example due to technical concerns. During a telephone appointment, body language cannot be used to help gauge patient understanding. Some research using questionnaire-based evaluation of patient preference and genetic counsellor experience did not identify differences between telephone and video appointments in patient reported distress or provision of genetic counsellor empathy [37]. Video appointments may be more advantageous for clinicians [37, 38], but technology access may mean that telephone consultations may be more acceptable to some patients.

It must also be acknowledged that the role of technology in assisting consultations is expanding, which may provide both challenges and opportunities. For example, automated conversational applications (chatbots) are being explored [39], and work is ongoing to explore the potential impact of these [40].

#### Workload management

It is important to ensure that workloads are managed appropriately with virtual consultations. Affording patient and clinician flexibility around appointments can be beneficial, but this may also lead to a range of other issues. Patients could join appointments from seemingly inappropriate locations, and expect clinicians to call back later. This could be mitigated by providing appropriate patient information before the appointment to manage patient expectations. Whilst on one hand it may be beneficial that the flexibility of virtual appointments makes it easier to schedule appointments at a mutually convenient time to clinician and patient, it is also important that the workload of the clinicians is managed appropriately. This would mean, for example, not lengthening the clinic schedule because of the travel time saved by VC. There is limited published literature on this area, but a recent prospective study analysing three healthcare specialties in the USA showed no increase in burnout for health professionals operating flexible scheduling and VC compared to a rigid, traditional scheduling pattern [41]. Healthcare professionals in this study from Rheumatology, Neurology and Paediatrics with flexible and virtual patient contact showed improved control over workload and reduced work-related stress after 6 months, assessed during the height of the pandemic [41].

Finally, keeping up to date and continuing professional development was a requirement during a global pandemic and remains so for practicing healthcare professionals. With the need to develop more online training tools during lockdowns when travel was not permitted, access to some remote resources became easier. There are training resources specific to improving virtual healthcare provision (https://www.westernstatesgenetics.org/telehealth-resources/) and these have potential to help workload management when accessed remotely.

## Conclusion

The COVID-19 pandemic imposed the rapid implementation of virtual consultations upon cancer genetic services– along with many other parts of the NHS. This has now introduced a wide range of opportunities and challenges.

There are opportunities to reduce geographical inequalities previously imposed by the location of clinics. There may be economic advantages and improved efficiency from virtual services. Cancer genetics services may be able to better meet patient expectations as the virtual world continues to expand into many aspects of life. Virtual consultations are kinder to the climate and present opportunities for further service development, such as seeing geographically separate family members together.

However, virtual healthcare processes can pose several significant challenges. The NHS needs to update and develop its existing IT infrastructure to ensure that it can consistently deliver a high standard of care via virtual clinics. Even with this, there are a range of levels of digital literacy and digital exclusion may be more pronounced in particularly vulnerable groups. Services must thus pro-actively work to maximise their accessibility, for example by accurately assessing whether digital literacy may be a concern on a patient-specific basis.

The experience of clinicians is also important to consider, and clinicians need to be equipped to feel confident delivering services virtually. Digital empathy is challenging, particularly when discussing difficult genetic testing results. It is imperative that clinicians become appropriately equipped to provide this type of empathetic response. Furthermore, virtual processes may involve inherent risks in terms of confidentiality and there may be concerns about coercion, which require clear patient information prior to the consultation, as well as the sensitivity and skills of the clinician.

The complexities in making clinical diagnoses mean that in-person appointments will always have a role, and clinical triage processes are crucial to navigating this balance. Considering the different challenges between video, telephone, and in-person consultations, the clinician’s insight and joint decision-making with the patient is necessary to determine which is most appropriate. Overall, the flexibility afforded by virtual consultations may be beneficial to both patients and clinicians, but clinician workload needs to be carefully managed.

In summary, the COVID-19 pandemic imposed the wide scale introduction of virtual consultations within cancer genetics services– along with many other parts of the NHS. Both the opportunities and the challenges of virtual care must be considered to ensure the appropriate, successful, and long-term implementation of virtual care in cancer genetic services.

## Data Availability

All references are included within the paper; no sources of primary data were used.
